# Using linear increment models for the imputation of missing composite outcomes in randomized trials

**DOI:** 10.1186/1745-6215-12-S1-A60

**Published:** 2011-12-13

**Authors:** Aidan G O’Keeffe, Daniel M Farewell, Brian DM Tom, Vernon T Farewell

**Affiliations:** 1 MRC Biostatistics Unit, IPH Forvie Site, Robinson Way, Cambridge, CB2 0SR, UK; 2 School of Medicine, Cardiff University, Neuadd Meirionnydd, Heath Park, Cardiff, CF14 4YS, UK

## 

In randomized trials, it is typical that a number of outcome variables are collected at each follow-up time. Sometimes, a composite outcome may be of scientific interest. A composite outcome is composed as a function of several patient-specific outcomes and could measure, for example, improvement or deterioration in the condition of a patient.

Multiple imputation is one method for handling patient drop-out in randomized trials, and usually involves a maximum likelihood-based model fitted to complete cases, which is then used to draw imputations for the missing data. In principle, imputation could be used to impute composite outcomes. However, composite outcomes may be complicated combinations of many outcome variables and, as a result, it can be difficult to impute composite outcomes, since they may not possess a distribution amenable to statistical modelling.

Using trial data on early rheumatoid arthritis patients, we examine the use of linear increments models, introduced by Diggle, Farewell and Henderson [1] as a basis for the multiple imputation of missing outcome information. These imputations are used to create random draws of the composite outcome ACR20, a binary indicator of disease improvement defined by the American College of Rheumatology. We compare the multiple imputation of ACR20 using linear increments methodology to that using more established maximum likelihood methods. We observe some evidence to suggest that the use of linear increment models may result in a more accurate imputation of missing ACR20 values. The methodology presented has broad applicability in randomized trials.
